# SPEARS: Standard Performance Evaluation of Ancestral haplotype Reconstruction through Simulation

**DOI:** 10.1093/bioinformatics/btaa749

**Published:** 2020-08-25

**Authors:** Heather Manching, Randall J Wisser

**Affiliations:** Department of Plant and Soil Sciences, University of Delaware, Newark, DE, 19716, USA; Department of Plant and Soil Sciences, University of Delaware, Newark, DE, 19716, USA

## Abstract

**Motivation:**

Ancestral haplotype maps provide useful information about genomic variation and insights into biological processes. Reconstructing the descendent haplotype structure of homologous chromosomes, particularly for large numbers of individuals, can help with characterizing the recombination landscape, elucidating genotype-to-phenotype relationships, improving genomic predictions and more. Inferring haplotype maps from sparse genotype data is an efficient approach to whole-genome haplotyping, but this is a non-trivial problem. A standardized approach is needed to validate whether haplotype reconstruction software, conceived population designs and existing data for a given population provides accurate haplotype information for further inference.

**Results:**

We introduce SPEARS, a pipeline for the simulation-based appraisal of genome-wide haplotype maps constructed from sparse genotype data. Using a specified pedigree, the pipeline generates virtual genotypes (known data) with genotyping errors and missing data structure. It then proceeds to mimic analysis in practice, capturing sources of error due to genotyping, imputation and haplotype inference. Standard metrics allow researchers to assess different population designs and which features of haplotype structure or regions of the genome are sufficiently accurate for analysis. Haplotype maps for 1000 outcross progeny from a multi-parent population of maize are used to demonstrate SPEARS.

**Availabilityand implementation:**

SPEARS, the protocol and suite of scripts, are publicly available under an MIT license at GitHub (https://github.com/maizeatlas/spears).

**Supplementary information:**

Supplementary data are available at *Bioinformatics* online.

## 1 Introduction

The genome is a mosaic of haplotype blocks that capture the evolutionary or breeding history of an individual. Reconstructing ancestral haplotype maps for populations is important for imputing untyped regions of the genome (Davies *et al.*, 2016), mapping quantitative trait loci (Mott *et al.*, 2000), investigating the recombination landscape (Morgan *et al.*, 2017) and inferring the evolutionary history and structure of haplotypes (Aylor *et al.*, 2011; Gabriel *et al.*, 2002). Accurately inferring the descendent structure of haplotypes is non-trivial, but several approaches for this have been developed: HAPPY (Mott *et al.*, 2000); MERLIN (Abecasis *et al.*, 2002); GAIN (Liu *et al.*, 2010); DOQTL (Gatti *et al.*, 2014); R/qtl2 (Broman *et al.*, 2019). These tools have been shown to perform well, but are mostly limited to specific population types or breeding schemes and can be computationally intensive with complex pedigrees or large numbers of markers.

Reconstructing Ancestry Blocks BIT by bit (RABBIT) is a flexible tool that uses a Markovian model to reconstruct ancestral haplotype maps for complex pedigrees involving various mating scenarios (Zheng et al., 2015). Compared to current tools, it has shown the highest accuracy for inbred lines from multi-parent pedigrees [including the mouse CC design (Churchill *et al.*, 2004) and *Arabidopsis thaliana* MAGIC design (Kover *et al.*, 2009)]. Here, tailored for RABBIT but extensible for other tools, we present a pipeline for the Standard Performance Evaluation of Ancestral haplotype Reconstruction through Simulation (SPEARS). SPEARS is designed to determine expectations for the accuracy of haplotype reconstruction software applied to user-defined populations. As proof-of-concept and a new demonstration of RABBIT, we develop a detailed picture on the variation in accuracy of genome-wide haplotype maps for progeny from a multi-parent population of maize.

## 2 Materials and methods

### 2.1 Simulation data

**Fig. 1.**
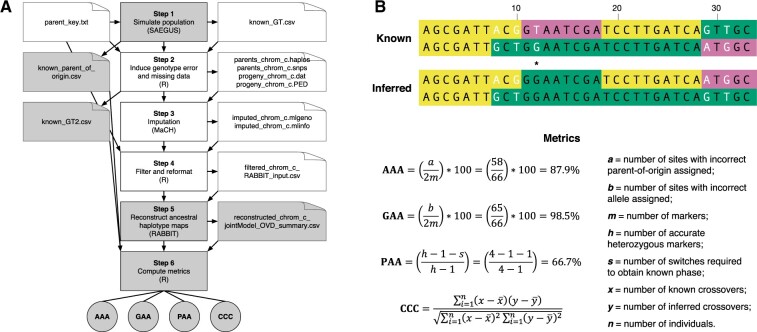
Overview of SPEARS. (**A**) Steps in the pipeline, file inputs/outputs and standard metrics. This begins with the creation of a simulated population (*n* virtual genotypes) using SAEGUS based on a user-provided population design, genetic map and parental genotype data. Genotyping errors and missing data are induced in the simulated data, which is then processed via MaCH (imputation) and RABBIT (ancestral haplotype reconstruction). Highlighted in gray are the main steps of the pipeline and files used to compute SPEARS metrics. (**B**) The figure portrays diplotypes (phased genotypes) for a genomic segment to describe how each summary statistic is calculated. Each of the three colors represents a distinct parent-of-origin. Heterozygous genotypes are shown in white text. The top diplotype represents known data that would be generated by simulation. The bottom diplotype represents inferred data that would be generated by RABBIT as part of the SPEARS pipeline. The formulas and variables used to calculate each metric are shown. CCC is computed as the correlation between known and inferred crossover events for all individuals and is therefore not shown for the example diplotype. The example shows five crossovers for the known individual and four crossovers for the inferred individual

This study introduces SPEARS (Fig. 1) which incorporates SAEGUS (https://github.com/maizeatlas/saegus) as a genome simulator to create known data. Custom R (R Core Team, 2017) scripts are used for data processing, metric calculations and graphical output. For proof-of-concept, we generated a virtual multi-parent outcross population of 1000 progeny (Supplementary Fig. S1). Real genotype data on inbred line parents of the population were used to initiate the simulation using 47 078 markers combined from genotyping-by-sequencing (GBS; Manching *et al.*, 2017) and the MaizeSNP50 BeadChip (Ganal *et al.*, 2011); filtered to remove residual heterozygous sites in the parents for compatibility with MaCH imputation (Li *et al.*, 2010). We also examined the impact of using fewer markers (23 584 GBS markers).

In SPEARS, to mimic analysis in practice, a user-defined global error rate is specified to induce genotyping errors and locus-specific missingness rates are specified to induce missing data for the virtual genotypes. SPEARS then uses MaCH for imputation (Li *et al.*, 2010), prior to reconstructing haplotype maps with RABBIT. For our use case, a global genotyping error rate of 0.006 was used based on trio analysis in prior work (Manching *et al.*, 2017). Missingness rates were also based on real data for the population that was simulated (column ‘F_MISS’ in Dataset S1). SPEARS reports the frequency of genotyping error and missing data per locus that is realized for the virtual population (e.g., see Supplementary Fig. S2 for the example population). Following imputation of the virtual genotypes, markers with imputation accuracy $R^{2}<0.8$ were filtered, resulting in 46 633 total markers (23 584 GBS markers) retained for RABBIT (Zheng *et al.*, 2015).

### 2.2 Reconstruction and evaluation of ancestral haplotype maps

To build haplotype maps for the simulated population the joint model of RABBIT is used to assign an optimal Viterbi path using the ‘origViterbiDecoding’ algorithm (Zheng *et al.*, 2015).

###  

Based on comparing known and inferred data per individual (Supplementary Fig. S3), SPEARS uses four metrics to assess different features of haplotype maps (Fig. 1B): (i) Ancestral Assignment Accuracy (AAA); (ii) Genotype Assignment Accuracy (GAA); (iii) Phase Assignment Accuracy (PAA); and (iv) Correlation between Crossover Counts (CCC). AAA is calculated as the proportion of markers that have the correct parent assigned on each homologue (given as a percentage). To calculate GAA, genotypes are assigned based on the inferred parent-of-origin and corresponding parental genotype data used as input, from which the proportion of genotype matches are calculated (given as a percentage). To assess phasing accuracy, PAA is calculated as the proportion heterozygous sites whose phase is correctly inferred relative to the previous heterozygous site (Lin *et al.*, 2002) and is calculated under the assumption that there are no genotyping errors and only among markers with correctly inferred genotype scores. AAA, GAA and PAA are averaged across all samples. The CCC is calculated as the correlation coefficient for the total number of crossovers across both homologues of all chromosomes per individual. Equations for each metric are shown in Figure 1B. For additional analysis, we also calculated parent certainty as the difference between the posterior probabilities of the two most likely parents at each marker (obtained from the ‘origPosteriorDecoding’ algorithm within RABBIT).

## 3 Results

SPEARS is designed to assess expectations for the accuracy of ancestral haplotype maps reconstructed with multiple software tools (imputation using MaCH and haplotype inference using RABBIT) for user-defined populations and genotype data (simulation using SAEGUS). Comparing known (simulated) and inferred (reconstructed) haplotype maps to compute the accuracy of different metrics (Fig. 1), SPEARS facilitates both genome-wide and regional appraisal of reconstructed haplotypes. Summary metrics reported by SPEARS are semi-independent (Supplementary Table S1) and describe separate features of common interest for haplotype analysis.

For demonstration, a use case was processed based on a real multi-parent population with prior estimates of genotyping error and missing data structure. Overall, SPEARS showed that highly accurate genome-wide haplotype maps could be generated from sparse genotype data (~1 marker per 50 kb) on an admixed non-inbred population (Supplementary Table S2). The average of genome-wide AAA per sample was 97.0%. Genomic regions with lower accuracy (minimum: 79.5%) showed decreases in parent certainty alone or in combination with a lower density of markers (Supplementary Figs S4 and S5), indicating identity-by-state among the parents and low marker density, but neither RABBIT nor the Viterbi algorithm *per se*, were main sources of error in the inference of parent-of-origin for haplotype blocks. Given the ancestral origin inferred at a marker locus, the corresponding parent genotype data is used to score the genotype for the inferred haplotype, to compute GAA. If the wrong parent-of-origin is inferred at a marker, but that parent shares the same genotype of the correct parent, GAA will be higher than AAA. We observed this for our use case (Supplementary Table S2), indicating that shared parental haplotypes contributed to inferring the incorrect parent-of-origin at 2.3% of the markers on average.

RABBIT performed very well in haplotype phasing with an average PAA of 99.4% across all samples. There was a high-positive CCC (*r *=* *0.87, *P *<* *2.2e-16) (Supplementary Fig. S6). However, the number of crossover counts per sample was downward biased with an average of 260 ± 16 versus 227 ± 14 for known and inferred results, respectively.

SPEARS can be used to guide decision making. For instance, genotyping platforms vary in cost and result in different marker densities, error rates and missingness structure. For the example population, reducing the marker density by half had essentially no effect on the quality of haplotype reconstruction (Supplementary Table S2). Genotyping error rates of 0.006 and 0.06 also showed little impact on the overall performance of haplotype reconstruction; however, for an error rate of 0.25 substantial reductions in performance were observed for AAA, GAA and CCC but not PAA (Supplementary Table S3).

## 4. Conclusion

Reconstruction of ancestral haplotypes from genomic data is useful for a number of applications. The use case presented here demonstrates how SPEARS estimates expectations for accuracy of the reconstruction process to guide investigators on the analysis of haplotype structure in multi-parent populations. It enables exploration of study designs before or after creating one. It can also be used to determine if certain features of haplotype data should be included/excluded in a study based on the accuracy of a corresponding metric, and the expectations can be reported. Moreover, one can assess whether specific chromosomes or regions of the genome, but not others, are sufficiently accurate for downstream analysis. Finally, as an extension, the genome simulator in SPEARS can model genetic architectures in order to test downstream analysis based on ancestral haplotype maps.

## Supplementary Material

btaa749_Supplementary_Data
