# Supplementary material for: SPEARS: Standard Performance Evaluation of Ancestral haplotype Reconstruction through Simulation
Source: Bioinformatics. 2020 Aug 25;37(6):868–70. doi: 10.1093/bioinformatics/btaa749 (PMC8097754; doi:10.1093/bioinformatics/btaa749)
Supplement: btaa749_Supplementary_Data [file btaa749_supplementary_data.zip › Manching_Wisser_SPEARS_Supplementary_Information.pdf]

Supplemental Information

SPEARS: Standard Performance Evaluation of Ancestral Reconstruction through Simulation

Heather Manching and Randall J. Wisser*

Department of Plant & Soil Sciences, University of Delaware, Newark, DE, USA
*Correspondence: rjw@udel.edu

Table of Contents

TABLES **2**

TABLE S1: CORRELATIONS BETWEEN SPEARS METRICS FOR THE EXAMPLE POPULATION. 2
TABLE S2. SPEARS RESULTS FOR LOW AND HIGH MARKER DENSITY. 3
TABLE S3. EFFECT OF GENOTYPING ERROR RATE ON SPEARS METRICS. 4

FIGURES **5**

FIGURE S1. MULTI-PARENT PEDIGREE USED TO DEMONSTRATE SPEARS. 5
FIGURE S2. FREQUENCIES OF GENOTYPING ERROR AND MISSING DATA REALIZED FOR THE EXAMPLE POPULATION. 6
FIGURE S3. ANCESTRAL HAPLOTYPE MAPS FROM SPEARS. 7
FIGURE S4. GENOME-WIDE ANCESTRAL ASSIGNMENT ACCURACY. 8
FIGURE S5. ANCESTRAL ASSIGNMENT ACCURACY FOR LOW AND HIGH MARKER DENSITIES. 9
FIGURE S6. CORRELATION AND DISTRIBUTIONS OF CROSSOVER COUNTS 10

Tables

Table S1: Correlations between SPEARS Metrics for the example population.

	AAA[a]	GAA[b]	PAA[c]	CO difference[d]
**AAA**	1.00	0.36	0.16	0.14
**GAA**	0.36	1.00	0.23	0.24
**PAA**	0.16	0.23	1.00	0.10
**CO difference**	0.14	0.24	0.10	1.00

[a]Ancestral assignment accuracy
[b]Genotype assignment accuracy
[c]Phase assignment accuracy
[d]Inferred crossover count minus known crossover count

Table S2. SPEARS Results for Low and High Marker Density.

Markers	AAA[a]	GAA[b]	PAA[c]	CCC[d]
n = 23,508	97.2% ± 0.6%	99.6% ± 0.1%	99.1% ± 0.2%	r = 0.89, p < 2.2 e-16
n = 46,633	97.0% ± 0.7%	99.3% ± 0.1%	99.4% ± 0.1%	r = 0.87, p < 2.2 e-16

[a]Ancestral assignment accuracy plus-minus 1 standard deviation.
[b]Genotype assignment accuracy plus-minus 1 standard deviation.
[c]Phase assignment accuracy plus-minus 1 standard deviation.
[d]Correlation between crossover counts.

Table S3. Effect of Genotyping Error Rate on SPEARS Metrics.

Error Rate[a]	AAA[b]	GAA[c]	PAA[d]	CCC[e]
0.006	97.2% ± 0.6%	99.6% ± 0.1%	99.1% ± 0.2%	r = 0.89, p < 2.2 e-16
0.06	96.4% ± 0.7%	99.3% ± 0.1%	99.0% ± 0.2%	r = 0.84, p < 2.2 e-16
0.25	60.0% ± 2.1%	84.5% ± 0.8%	98.8% ± 0.2%	r = 0.49, p < 2.2 e-16

[a]Analysis based on 23,584 GBS markers.
[b]Ancestral assignment accuracy plus-minus 1 standard deviation.
[c]Genotype assignment accuracy plus-minus 1 standard deviation.
[d]Phase assignment accuracy plus-minus 1 standard deviation.
[e]Correlation between crossover counts.

Figures

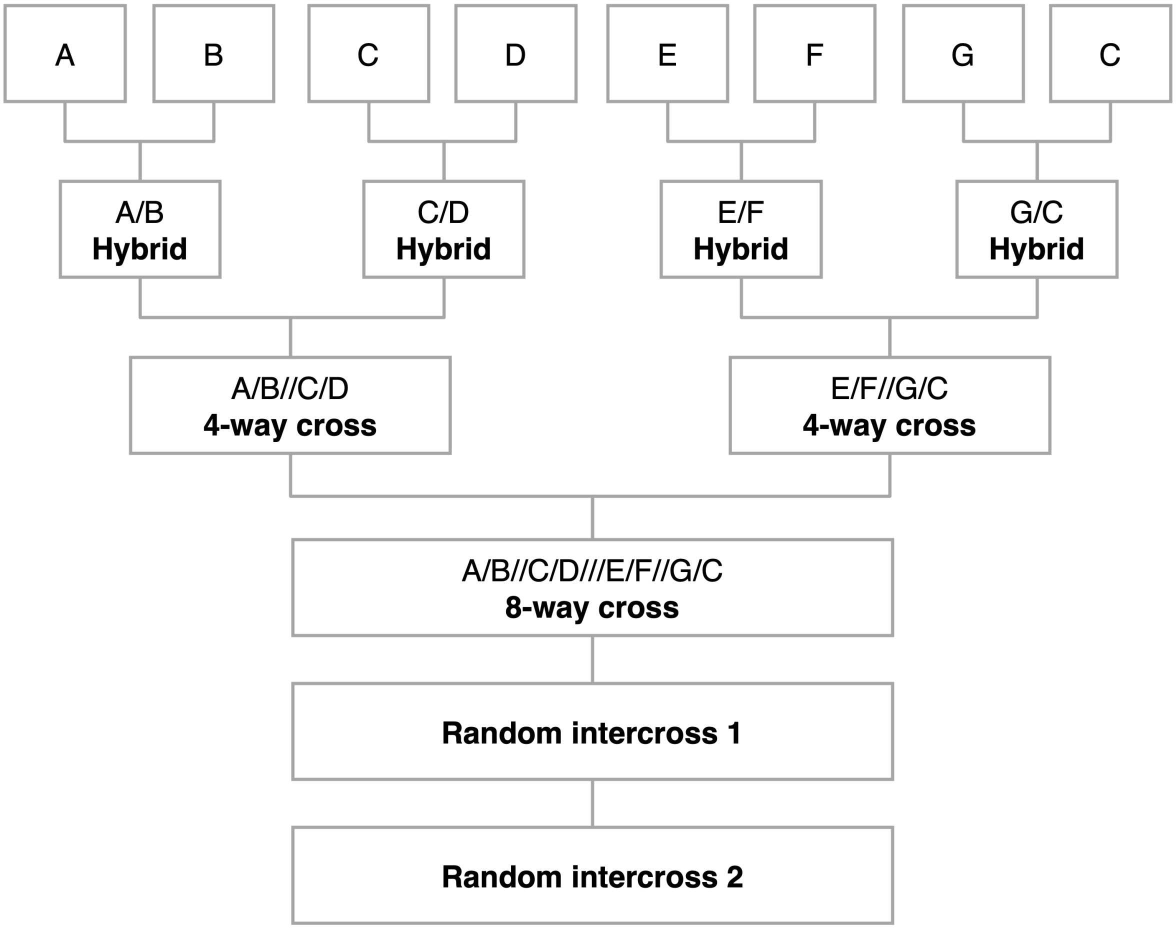

Figure S1. Multi-Parent Pedigree used to Demonstrate SPEARS. As proof-of-concept for the study, mimicking the pedigree of a real population of maize, multiple inbred line parents were used to create virtual genotypes (parent C was used twice). Each generation was created by randomly intermating according to the pedigree. One-thousand progeny from the last intercross were used for SPEARS. See the code for this example at github (https://github.com/maizeatlas/spears/blob/master/1_SAEGUS.py) for detailed parameters used for the simulation in SAEGUS.

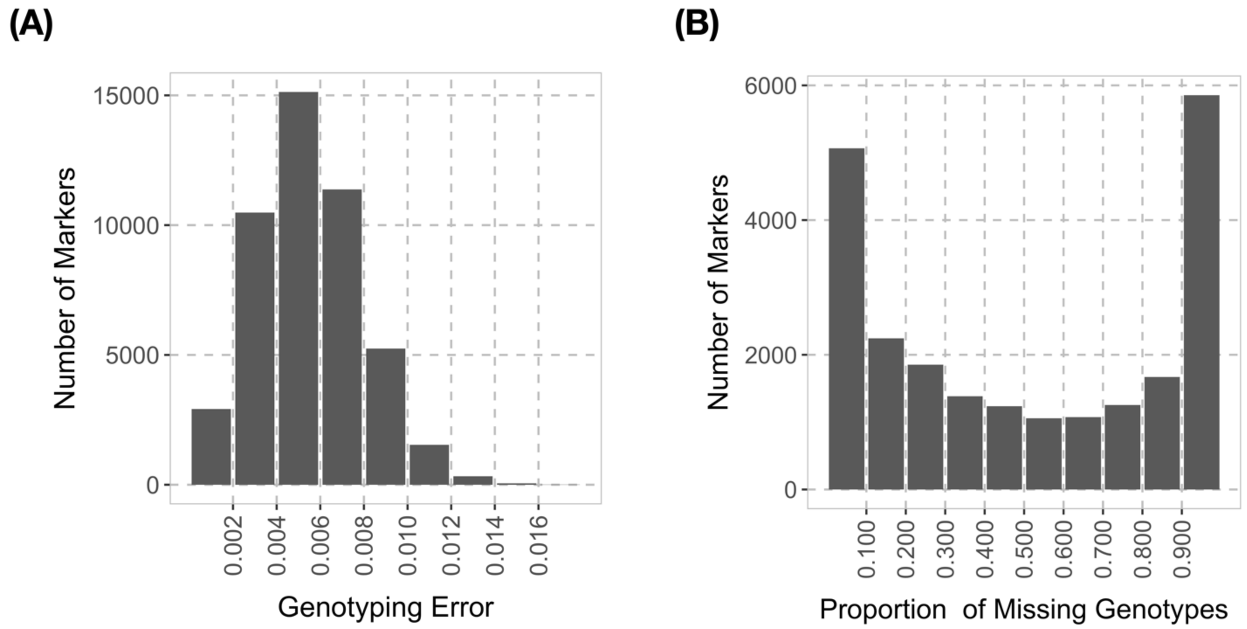

Figure S2. Frequencies of Genotyping Error and Missing Data Realized for the Example Population. A) Genotyping error distribution. The proportion of individuals with an induced genotyping error is shown across all markers. B) Missing data distribution. Only markers that did not have completely missing data are plotted (22,689 out of 47,078).

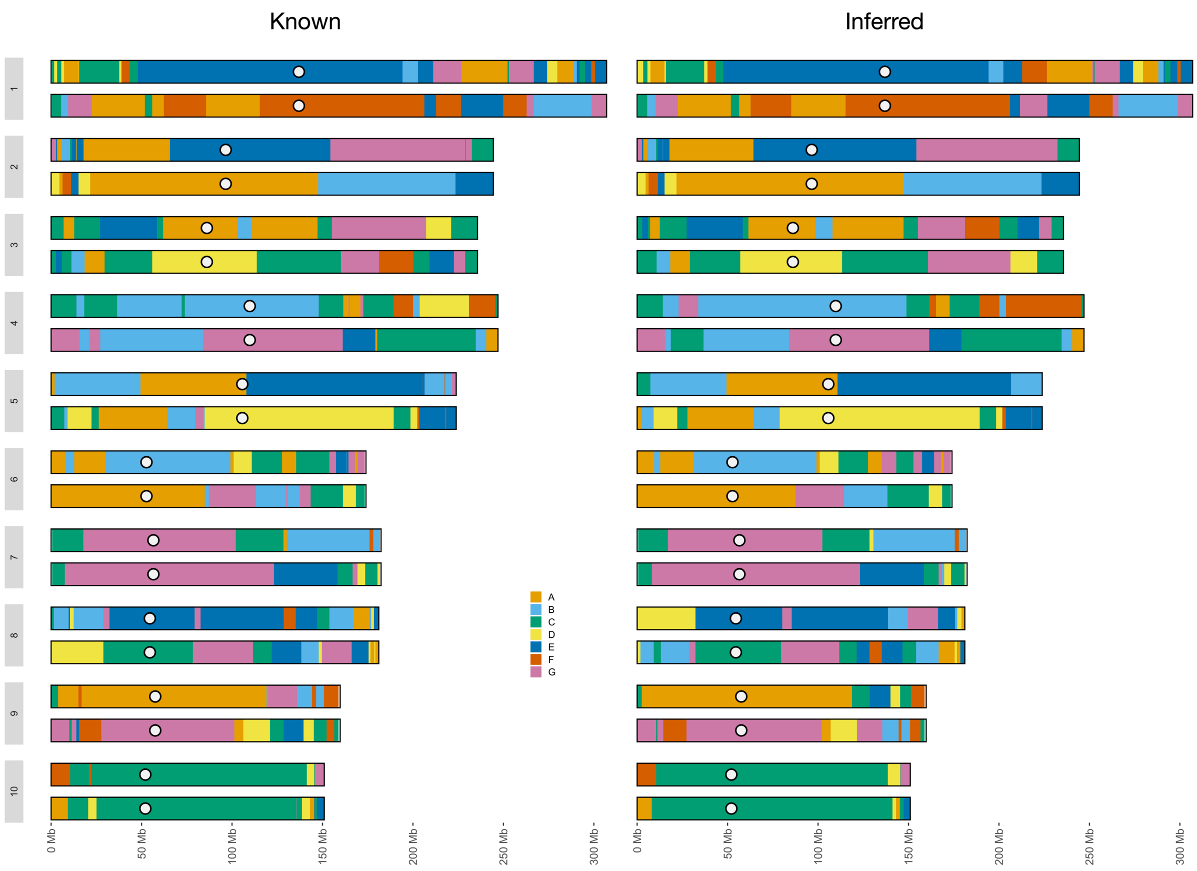

Figure S3. Ancestral Haplotype Maps from SPEARS. The known (simulated) and inferred (RABBIT) haplotype map for all chromosomes in a single individual is shown. Colors correspond to the seven founding parents of the admixed population (Figure S2). The centromere is represented as a white circle.

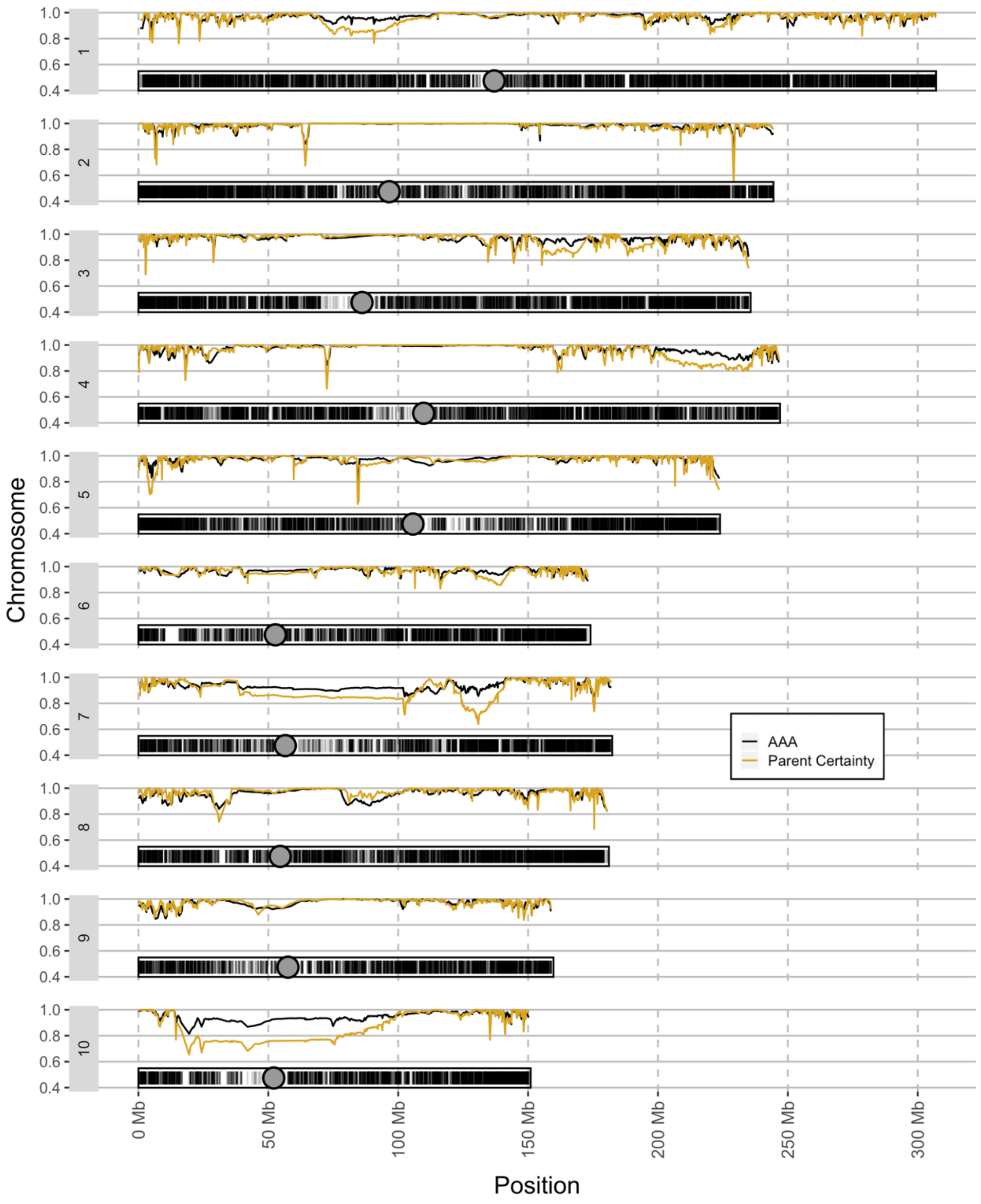

Figure S4. Genome-wide Ancestral Assignment Accuracy. AAA (black), parent certainty (gold), marker density (rug along x-axis), and centromere position (grey dot with black outline) were plotted for each of the ten chromosomes of maize. Parent certainty is the difference in parent probabilities for the two most likely parents at each marker, where a higher value indicates more certainty in assignment of the parent (i.e., a larger difference between the two most likely parents).

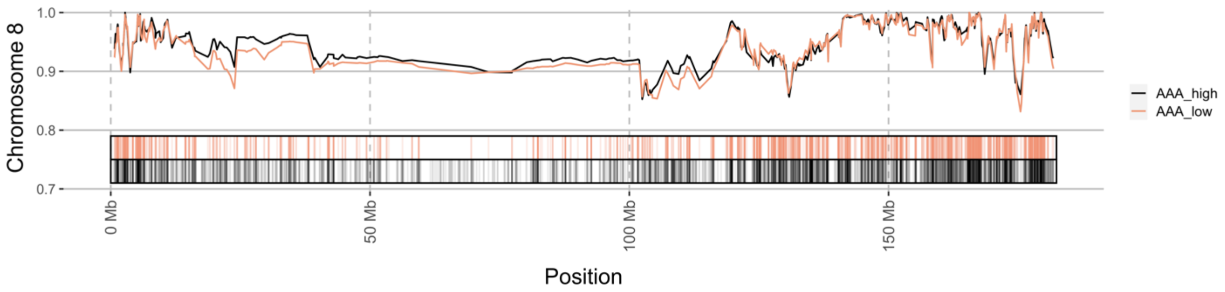

Figure S5. Ancestral Assignment Accuracy for Low and High Marker Densities. AAA is shown for SPEARS using overlapping marker sets between analyses using *n* = 23,508 markers (gold) and *n* = 46,633 markers (black) along with the corresponding marker densities (rugs along x-axis) for chromosome 8. The region between 0 Mb and 50 Mb demonstrates an increase in accuracy in relation to marker density. The region between 100 Mb and 150 Mb demonstrates minimal change in accuracy despite an increase in marker density and is likely due to IBS/IBD.

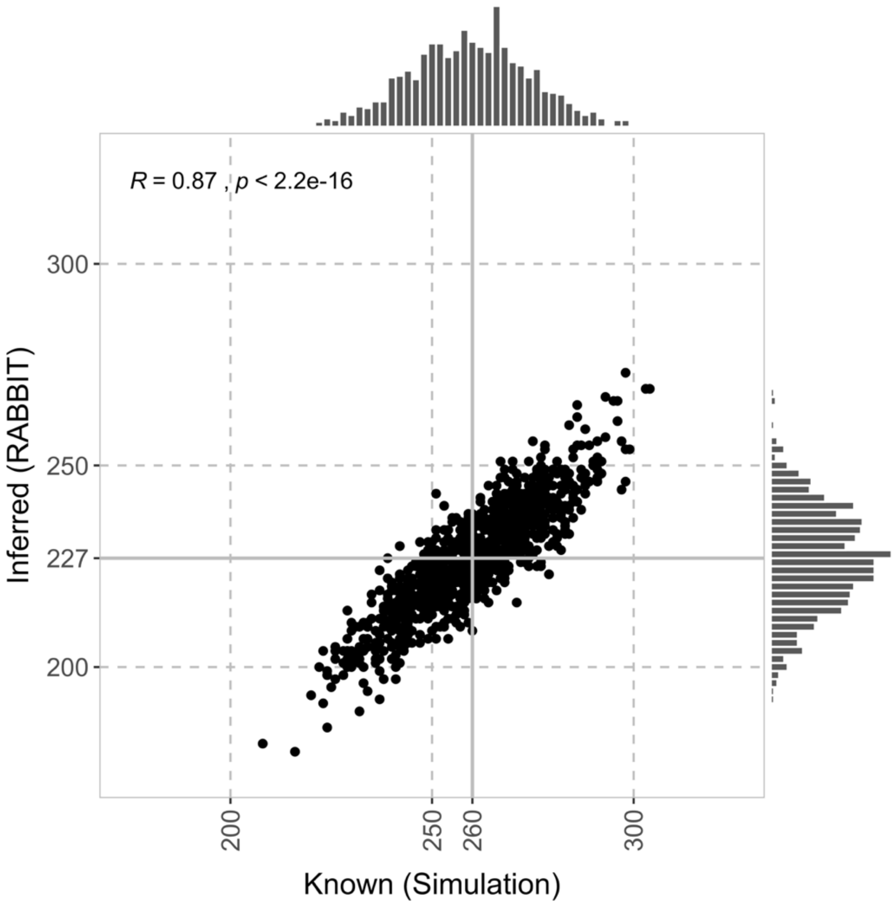

Figure S6. Correlation and Distributions of Crossover Counts Each point is the number of crossovers for a given individuals known versus inferred value. Solid grey lines indicate the average number of crossovers among individuals. Marginal histograms show the distributions in crossover counts.
